# Sequence Variants in PSMB8/PSMB9 Immunoproteasome Genes and Risk of Urothelial Bladder Carcinoma

**DOI:** 10.7759/cureus.36293

**Published:** 2023-03-17

**Authors:** Nasser A Elhawary, Samar N Ekram, Iman S Abumansour, Zohor A Azher, Imad A AlJahdali, Najiah M Alyamani, Hind M Naffadi, Ikhlas A Sindi, Abdulaziz Baazeem, Anmar M Nassir, Ahmad H Mufti

**Affiliations:** 1 Medical Genetics, Umm Al-Qura University, Mecca, SAU; 2 Community Medicine, Umm Al-Qura University, Mecca, SAU; 3 Molecular Genetics, University of Jeddah, Jeddah, SAU; 4 Biology, King Abdulaziz University, Jeddah, SAU; 5 General Surgery, Umm Al-Qura University, Mecca, SAU; 6 Surgery, Umm Al-Qura University, Makkah, SAU

**Keywords:** protein-protein interactions, statistial analysis, taqman genotyping assay, molecular carcinogenesis, functional enrichment analysis, saudi population, genotyping, psmb8/psmb9, immunoproteasome subunits, urothelial bladder carcinoma

## Abstract

Background: The *PSMB8* and *PSMB9 *immunoproteasome* *genes are essential in cell processes, such as decisions on cell survival or death, the cell cycle, and cellular differentiation. Because recent evidence has demonstrated an immunological role for proteasomes in various malignancies, including urothelial bladder carcinoma (UBC), we evaluated single nucleotide polymorphisms (SNPs) in *PSMB9* and *PSMB8*. We determined any associations between these SNPs and susceptibility to UBC in the Saudi community.

Methods: Samples of genomic DNA were taken from buccal cells of 111 patients with UBC and 78 healthy controls. TaqMan Real-Time PCR was used to determine genotype distributions and allele frequencies for the *PSMB9* rs17587 G>A and *PSMB8* rs2071543 G>T SNPs. We used SNPStats (https://www.snpstats.net) to choose each SNP's best interactive inheritance model.

Results: The *PSMB9* rs17587 SNP was associated with the risk of UBC (odds ratio [OR] = 5.21, *P* < 0.0001). In contrast, the *PSMB8* rs2071543 SNP showed no association with UBC risk (OR = 1.13, *P* = 0.7871). In terms of genotypic distribution, the rs17587 G>A SNP was more frequent in UBC cases than controls in both the dominant (OR = 7.5; 95% confidence interval, 3.7-15.1; *P* = 0.0051) and recessive (OR = 17.11, 95% confidence interval 5.1-57.4; *P* = 0.0026) models. Genotypic distribution of the *PSMB8* rs2071543 G>T SNP was not significantly different between cases and controls in any interactive inheritance models (*P* > 0.05).

Conclusion: These results suggest a potential role for *PSMB9* as a biomarker for increased UBC risk. Discovering more genetic variants within immunoproteasome genes related to antigen presentation could help further our understanding of this risk.

## Introduction

The International Cancer Genome Consortium (ICGC) and The Cancer Genome Atlas (TCGA) research network has generated a set of 24 papers analyzing whole cancer genomes and transcriptomic data from 38 tumor types [1](Consortium ITP-CAoWG, 2020). Bladder carcinoma (BC) is the fourth most common tumor in developed countries, characterized by a high mutation rate and many neoplastic antigens [2]. The general five-year survival rate for people with BC is 77% (https://www.cancer.net/cancer-types/bladder-cancer/statistics), and an estimated 212,536 people died from BC worldwide in 2020. Although mortality rates for BC decreased by almost 2% from 2015 to 2019 (https://www.cancer.net/) after decades of no significant change, they decreased by 20-40% for breast, prostate, colorectal, and lung cancers. 

Almost 83% of all diagnosed BC is urothelial bladder carcinoma (UBC, OMIM 109800). There have been no significant improvements in treating metastatic UBC in the past 30 years, and chemotherapy is still the management standard [3]. Approximately 70-80% of BC patients are diagnosed with non-invasive UBC (pTa-pT1), and the remaining patients have invasive UBC that often recurs [4]. The estimated recurrence rate within five years of transurethral resection of bladder tumors (TURBT) is 75%; Because of these high rates of metastasis and recurrence, the five-year survival rate for patients with UBC is about 57% [5].

The selection of candidate genes is always challenging, especially for cancers and immune-associated disorders that involve endogenous toxins, environmental risk factors, and gene-gene interactions. Recently, some evidence has demonstrated the immunological roles of proteasomes in various malignancies [6]. Proteasomes are significant components of the ubiquitin-proteasome system, including a cascade of E1, E2, and E3 enzymes [7] that mediate activation, conjugation, and ligation of the small ubiquitin protein to substrate proteins in the cytoplasm that the proteasome then degrades into smaller peptides [8]. Instead of permanently destroying all targeted proteins, proteasomes may regulate the functional activation of some of these proteins. The proteasome is integrated into virtually all cell processes, including decisions on cell survival or death, the cell cycle, and differentiation [6], thus serving as a regulator of carcinogenesis. Increased proteasome activity implies greater production and assembly of catalytically active proteasome complexes [9]. 

The transporter associated with antigen processing (TAP), a pump that translocates peptides degraded by proteasomes from the cytoplasm to the endoplasmic reticulum, was discovered in a study related to antigen presentation [10]. In the proximity of TAP genes, the PSMB9 (LMP2, OMIM 177045) and PSMB8 (LMP7, OMIM 177046) genes can be induced by interferon-γ treatment, leading to the transcription of three distinct proteasome subunits with altered catalytic characteristics [8]; the resulting proteasome (i.e., second isoform) is known as an immunoproteasome (i-proteasome).

The cascade peptides generated by i-proteasomes positively correlate with antigen presentation mediated by the major histocompatibility complex (MHC)-1, essential for activating the immune system [8]. The immune system could be applied to recognize and destroy cancer cells [11] (. Expression profiling has revealed that PSMB8 mRNA is expressed in relatively low levels in the bone marrow, muscle, and testis and much higher levels in the bladder, lung, and spleen (P < 0.05) [11]. Hence, Chen et al. [12] concluded that PSMB8 signifies favorable overall survival [13] and disease-specific survival in UBC, invasive breast carcinoma, ovarian serous cyst adenocarcinoma, mesothelioma, and cutaneous skin melanoma and may therefore function more in a protective role. Conversely, high expression of PSMB8 was associated with poor survival in acute myeloid leukemia and lung adenocarcinoma, pancreatic adenocarcinoma, and uveal melanoma [12]. In anti-tumor immunity mediated by T cells, the overexpression of PSMB8 was reported to reduce colony formation after radiation, with a significant increase in the expression of apoptosis-inducing molecules such as cleaved PARP and cleaved caspase-3 [14].

The PSMB9 gene encodes one of the catalytic subunits of the i-proteasome, which replaces the standard catalytic subunit in the 20S core during proteasome assembly; PSMB9 engages in macromolecular catabolic processes involving proteasomes and may limit antigen processing and presentation, making the PSMB9 gene an attractive candidate gene for raising cancer risk [15].

Despite being essential in cellular and immunological functions, the role of PSMB8 and PSMB9 has not been determined in all types of cancers, as highlighted in at least 78 publications [16], including 19 meta-analyses [17]. Few molecular genetics studies have focused on relationships between these i-proteasome genes and bladder cancer [18-20]. However, despite these studies, research on PSMB8/PSMB9 loci concerning genetic risks in bladder cancers still needs to be completed. Hence, in the present study, we evaluated the PSMB8 rs2071543 G>T (p.Q49K) and PSMB9 rs17587 G>A (p.R60H) variant alleles, their possible association with UBC and the influence of various genotypes on the risk and severity of the disease.

## Materials and methods

Study population

**Figure 1:**
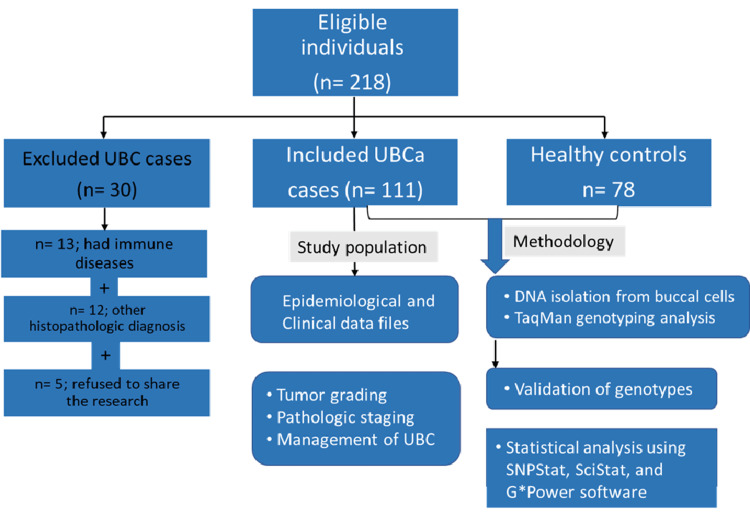
Flow chart of study inclusion, exclusion, and applied methodology. Abbreviations: UBC, urothelial bladder carcinoma

The study included 111 Saudi patients diagnosed with UBC admitted to the Urology Department at King Abdullah City Hospital in Mecca between June 2017 and January 2021. Gender, age at diagnosis, family history of cancer, cigarette smoking habits, alcohol intake, tumor grades, pathologic grades (i.e., pTa, pT1, pT2), and metastases were all recorded as epidemiologic and clinical factors for statistical analysis. Invasive bacillus Calmette-Guerin (BCG), immunotherapy, conservative TURBT-BCG therapy (by injection), chemotherapy, chemo-radiotherapy, and radical cystectomy had been used to treat the disease, depending on the case. Patients with a history of cancer, radiation, chemotherapy, or metastasized tumors from unknown sources were excluded from the study. Patients with immunological diseases or any histopathologic diagnosis besides UBC were excluded. Regular follow-up appointments at governmental hospitals in Mecca recruited 78 healthy individuals with no evident clinical characteristics of malignancies or immunological disorders as controls (Figure 1).

TaqMan genotyping

DNA samples were extracted from the buccal mucosa of all individuals using the Oragene.DNA-OGR-575 kit (DNA Genotek Inc., Ottawa, ON, Canada) with some modifications. Briefly, whole buccal cells were lysed in the OGR buffer at 53°C. The liberated DNA was then precipitated by ethanol and dissolved in an elution buffer [21]. We applied TaqMan Real-Time PCR (Thermo, Applied Biosystems, USA) to genotype individuals for the PSMB8 rs2071543 'p.Q49K' (C__15869253_10) and PSMB9 rs17587 'R60H' (C___8849004_1_) SNPs using a 7500 Fast-Dx Real-Time PCR System (Thermo Fisher Scientific, USA) with the transcript accessions and protein IDs NM_002800 (NP_000584.2) and NM_002800.5 (NP_002791.1), respectively, on the current human reference genome assembly (i.e., GRCh38). The probe sequences were 5`-AGG GGC TTC CCT ACT GCC CCG ACC T[G/T]C ATT CCC CGG GGT AAA GCG AGC TCT-3` for PSMB8 rs2071543G>T and 5`-GAC AAG CTG TCC CCG CTG CAC GAG C[A/G]C ATC TAC TGT GCA CTC TCT GGT TCA-3` for PSMB9 rs17587G>A. We used VIC/FAM dyes as the reporter/quencher probes. All DNA samples and negative controls were reassessed in duplicate.

Statistical analysis

We examined Hardy-Weinberg equilibrium (HWE) for the PSMB8 rs2071543 and PSMB9 rs17587 SNPs in all individuals (http://www.oege.org/software/hwe-mr-calc.shtml). SciStat (https://www.scistat.com/statisticaltests/likelihoodratios.php) software was used to evaluate likelihood ratios, Odds ratios (ORs), confidence intervals (CIs), and one-way Chi-squared tests related to tumor grades, pathologic stages, clinical management, and recurrences. However, the likelihood ratio was proper to evaluate the post-test odds from the pre-test odds of disease. We used SNPstats software to choose the best inheritance model (i.e., codominant, dominant, recessive, or overdominant) for each SNP based on a low Akaike information criterion (AIC). The haplotyping analysis and the extent of linkage disequilibrium between SNPs were calculated regarding the coefficient of linkage disequilibrium (D`) and the correlation coefficient between pairs of loci (r). We used G*Power software (Franz Faul, Universität Kiel, Germany ver. 3.1.9.2) (http://www.psycho.uni-duesseldorf.de/abteilungen/aap/gpower3/download-and-register/) to perform a priori power analysis to calculate sufficient sample sizes to achieve an adequate power sensitivity of 80% and a criterion probability of 𝛼 = 0.05.

Protein-protein interaction network

Using the STRING database (https://string-db.org), the potential protein-protein interaction (PPI) network was analyzed to predict functional interactions between PSMB8/PSMB9 and other proteins. 

Enrichment analysis of PSMB8/PSMB9

We used DAVID tools (https://david.ncifcrf.gov/) to identify enriched biological themes, including gene ontology (GO) and Kyoto Encyclopedia of Genes and Genomes (KEGG) pathways. A false discovery rate (FDR) < 0.05 and gene counts ≥ 3 in GO annotation analysis, as well as *P* < 0.05 and gene counts ≥ 40 in KEGG pathway enrichment analysis, were considered statistically significant.

## Results

Clinico-epidemiologic population profile 

**Figure 2:**
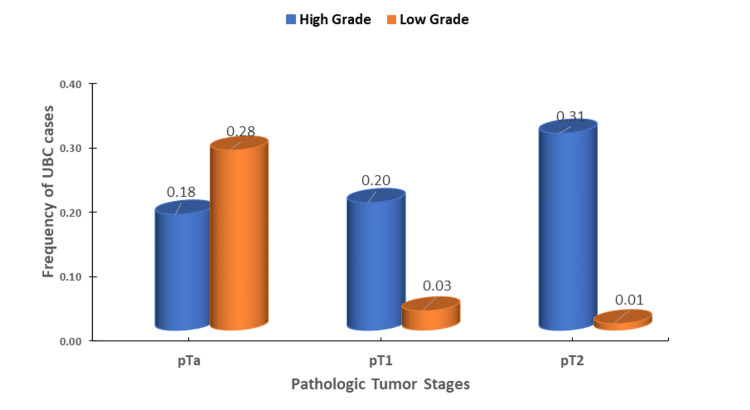
Tumor grades and pathological stages among patients with urothelial bladder carcinoma UBC: Urothelial bladder carcinoma

For the study, 111 eligible Saudi individuals with UBC (103 men and 8 women; ratio, 12.9: 1) and 78 healthy controls (73 men and 5 women; ratio, 14.6: 1) were enrolled. The mean age of patients was 61.8 ± 10.63 years (range 64-90 years), with no significant difference between cases and controls (P > 0.05). There was also no significant difference between cases and controls in the proportion who currently smoked cigarettes (80.8% versus 76.9%, respectively; P = 0.6). The clinical characteristics of UBC cases are shown in Table 1 and Figure 2. A significant difference was found in the proportion of cases with high-grade versus low-grade tumors (68.5% versus 31.5%; χ2 = 15.1, P = 0.0001). Regarding the pathologic stage, 46% of tumors were pTa, 22.5% were pT1, and 31.5% were pT2. The likelihood ratios of the pathologic stages were 1.7 for pTa (95% CI, 1.27-2.29) and 0.7 for pT2 (95% CI, 0.66-1.28) with a significant difference among these stages (χ2 = 9.30, P = 0.0096). There was also a statistical difference among choices for disease management (χ2 = 199.4; P < 0.0001), with likelihood ratios of 9.5 (95% CI, 5.7-15.7) for BCG therapy and 7.6 (95% CI, 4.9-11.9) for conservative TURBT-BCG therapy. Most cases (77/111, 69.4%) showed no disease recurrences (z = 6.6, 95% CI, 4.69-9.27).

**Table 1:** Clinical characteristics in UBC cases *Not significant, **significant; ***highly significant. Abbreviations: BCG, *bacillus Calmette-Guerin*; CI, confidence interval; TURBT, transurethral resection of bladder tumor.

Characteristic	Description	No. of cases (%)	Likelihood ratio	χ^2^ (P value)
			(95% CI)	
Tumor grade	Low grade	35 (31.5)	0.46 (0.34-0.62)^*^	15.1 (0.0001)^***^
	High grade	76 (68.5)	2.17 (1.6-2.94)^**^	
Pathologic stage	pTa	51 (46.0)	1.71 (1.27-2.29)^**^	9.30 (0.0096)^**^
	pT1	25 (22.5)	0.61 (0.40-0.85)^*^	
	pT2	35 (31.5)	0.73 (0.66-1.28)^*^	
Disease management	BCG	85 (76.6)	9.52 (5.7-15.67)^***^	199.4 (<0.0001)^***^
	TURBT-BCG	90 (81.1)	7.64 (4.9-11.87)^***^	
	Radical cystectomy	11 (9.9)	0.23 (0.11-0.36)^*^	
	Chemo-radiotherapy	6 (5.4)	0.10 (0.05-0.23)^*^	
	Chemotherapy	4 (3.6)	0.07 (0.03-0.18)^*^	
Number of recurrences	1	5 (4.5)	0.15 (0.06-0.36)^*^	8.9 (0.0118)^**^
	2	19 (17.1)	0.60 (0.39-0.94)^*^	
	3	10 (9.0)	0.29 (0.16-0.53)^*^	

Hardy-Weinberg equilibrium and power analysis

In cases versus controls, the observed genotype distributions were consistent with HWE for the PSMB9 rs17587 G>A SNP (χ2 = 0.008; P = 0.9272 versus χ2 =1.28; P = 0.2580, respectively) but deviated from HWE for the PSMB8 rs2071543 G>T SNP (χ2 = 71.1; P = 0.000 versus χ2 = 49.1; P = 0.000, respectively). The statistical priori power analysis found that a power of 80% would require 5,253 cases and 3,677 controls for the PSMB8 rs2071543 SNP and 30 cases and 21 controls for the PSMB9 rs17587 SNP, which seemed unachievable during a limited period. Thus, the post hoc statistical analysis for the rs2071543 and rs17587 SNPs revealed actual powers of 6.89% and 99.98%, respectively, among our 189 participants (111 cases and 78 controls).

Allelic frequencies of *PSMB8/PSMB9* loci

As shown in Table 2, the ORs of the allelic variants were 5.2 (95% CI, 3.32-8.23; P < 0.0001) for PSMB9 rs17587 G>A and 1.13 (95% CI, 0.75-1.70; P = 0.79) for PSMB8 rs2071543 G>T. For PSMB9 rs17587, the A-allele was about three times more frequent in cases than in controls (64% versus 25%, respectively). In contrast, no significant difference was found in the frequency of the variant T-allele in the PSMB8 rs2071543 G>T SNP in cases versus controls (47% versus 44%, respectively). The ORs of the allelic variants were 5.2 (95% CI, 3.32-8.23; P < 0.0001) for PSMB9 rs17587 and 1.13 (95% CI, 0.75-1.70; P = 0.787) for PSMB8 rs2071543 G>T.

**Table 2:** Genotype distributions and allele frequencies of PSMB9 and PSMB8 variants in UBC cases and controls (adjusted by age) Abbreviations: AIC, Akaike information criterion; CI: confidence interval; H, histidine; K, lysine; OR, odds ratio; Q, glutamine; R, arginine; UBC, urothelial bladder carcinoma.

Genetic model	Interactive	No. of cases (%)	No. of controls (%)	OR (95% CI)	P value^a^	AIC
	genotype	n = 111	n = 78			
PSMB9 rs17587 G>A (p.R60H)						
Genotype distribution:						
Codominant	G/G	15 (13.5)	42 (53.9)	1		
	G/A	51 (15.4)	33 (42.3)	0.3 (0.07-1.06)	0.0017	68.2
	A/A	45 (40.5)	3 (3.8)	0.1 (0.00-0.35)		
Dominant	G/G	15 (13.5)	42 (53.9)	1		
	G/A-A/A	96 (86.5)	36 (46.1)	7.5 (3.70-15.1)	0.0051	71.1
Recessive	G/G-G/A	66 (59.5)	75 (96.2)	1		
	A/A	45 (40.5)	3 (3.8)	17.1 (5.1-57.4)	0.0026	69.9
Overdominant	G/G-A/A	60 (54.0)	45 (57.7)	1		
	G/A	51 (46.0)	33 (42.3)	1.2 (0.7-2.07)	0.8892	78.9
Allele frequency:						
	G	81 (0.36)	117 (0.75)	1		
	A	141 (0.64)	39 (0.25)	5.2 (3.32-8.23)	< 0.0001	NA
PSMB8 rs2071543 G>T (p.Q49K)						
Genotype distribution:						
Codominant	G/G	9 (8.1)	9 (11.5)	1		
	G/T	99 (89.2)	69 (88.5)	1.4 (0.54-3.80)	0.4672	77.3
	T/T	3 (2.7)	0 (0.0)	7.0 (0.32-154)	0.218	76
Dominant	G/G	9 (8.1)	9 (11.5)	1		
	G/T-T/T	102 (91.9)	69 (88.5)	1.4 (0.56-3.9)	0.431	77
Recessive	G/G-G/T	87 (97.3)	78 (100)	1		
	T/T	3 (2.7)	0 (0.0)	6.3 (0.32-123.5)	0.227	76.5
Overdominant	G/G-T/T	12 (10.8)	9 (11.5)	1		
	G/T	99 (89.2)	69 (88.5)	1.1 (0.43-2.7)	0.876	78.3
Allele frequency:						
	G	117 (0.53)	87 (0.56)	1		
	T	105 (0.47)	69 (0.44)	1.13 (0.75-1.70)	0.787	--

Genotypic distribution of *PSMB8/PSMB9* loci

Also shown in Table 2, the genotypic distribution of the PSMB9 rs17587 SNP differed significantly between cases and controls under the recessive model of inheritance that corresponded to minimum AIC value (OR = 17.11; 95% CI, 5.1-57.4; P = 0.0026). The combined G/A+A/A genotype in the rs17587 SNP was more frequent in cases than controls under the dominant model (86.5% versus 46.1%), and the A/A genotype was more frequent in cases than controls in the recessive model (40.5% versus 3.8%). The wild-type G/G genotype was increased in controls, and the variant A/A genotype was significantly more prevalent in UBC patients (P < 0.0001), implicating this amino acid change in UBC risk. In contrast, the dominant model was the best inheritance model for the PSMB8 rs2071543 SNP. Although rs2071543 G/T was more frequent in cases than controls, the difference was not statistically significant (OR = 0.40; 95% CI, 0.06-2.03; P = 0.2521). The rs2071543 T/T genotype was rare in cases (2.7%) and absent in controls.

Haplotype analysis and linkage disequilibrium

The results of haplotype analysis are presented in Table 3. Among the four possible haplotypes of the PSMB9 rs17587 (R60H) and PSMB8 rs2071543 (Q49K) loci, the H-K haplotype was significantly more frequent (24.28%) than the R-Q haplotype (OR = 0.19; 95% CI, 0.01-1.51; P = 0.035). The overall haplotype distribution differed significantly between cases and controls (P = 0.0024), and there was no linkage disequilibrium between the PSMB9 rs17587 and PSMB8 rs2071543 SNPs (D`= 0.0536, r = 0.0519, r2 = 0.0027; P = 0.5599).

**Table 3:** Haplotype distributions (n = 189, adjusted by age) Abbreviations: CI, confidence interval; H, histidine; K, lysine; OR, odds ratio; Q, glutamine; R, arginine.

Haplotype	rs17587G>A	rs2071543G>T (Q49K)	Frequency	OR (95% CI)	P value
	(R60H)				
1	R	Q	0.3063	1	---
2	H	K	0.2428	0.09 (0.01-1.01)	0.045
3	H	Q	0.2333	0.07 (0.01-0.78)	0.034
4	R	K	0.2175	0.13 (0.01-2.66)	0.19
Global haplotype association (P value = 0.0024)					

Protein-protein interaction network

**Figure 3:**
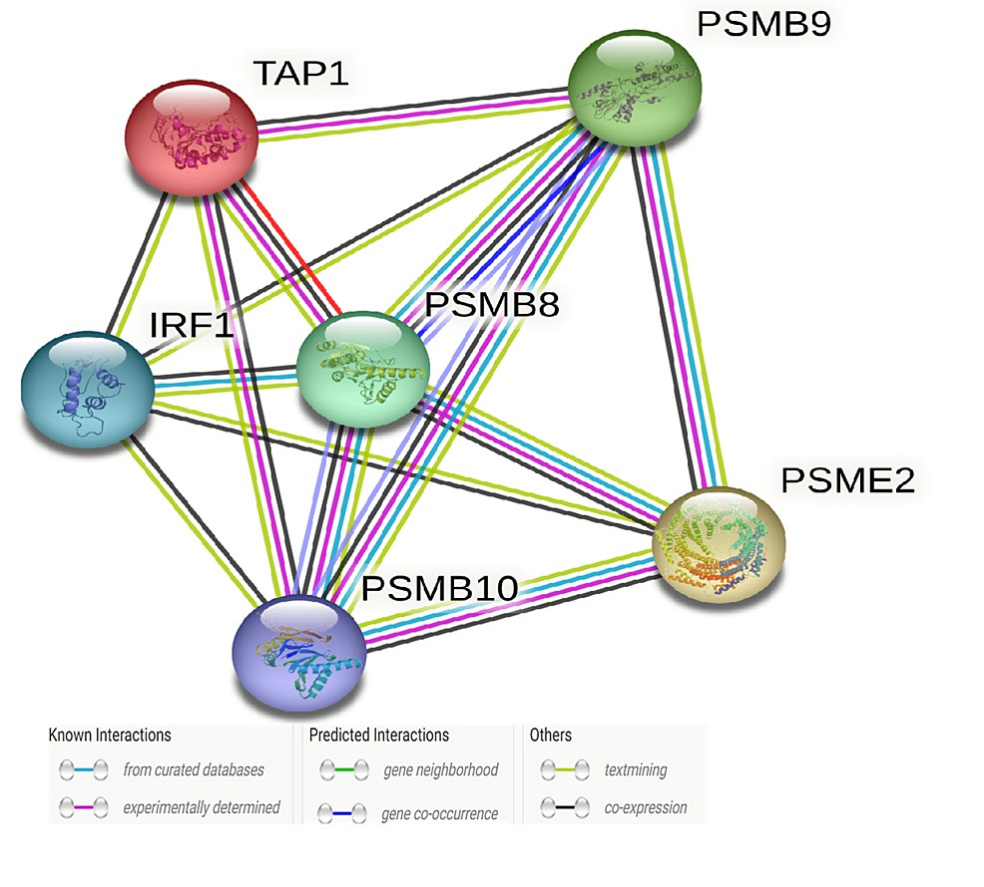
Protein-protein interactions predicted by STRING (https://string-db.org/). Strong interactions were predicted between PSMB8/PSMB9 and co-expressed TAP1, PSMB10, PSME2, IRF1. Colored nodes (n = 6) represent proteins and the first shell of interactors (average node degree = 4.67). Edges represent specific and meaningful protein-protein associations (n = 14) (i.e., proteins jointly contribute to a shared function).

The results of STRING analysis documented nine proteins (nodes) and 26 associations (edges) present in the PPI network of PSMB8/PSMB9 (Figure 3). Significantly more interactions were predicted than expected for a random set of proteins of the same size and degree of distribution (P < 1.05e-8).

Functional enrichment analysis

Table 4 highlights the functional enrichment of PSMB8/PSMB9 and related proteins in biological processes, including the proteasome ubiquitin-independent protein catabolic process (GO:0010499), cellular components, including the proteasome core complex and alpha/beta subunits (GO:0019773/GO:0019774), Reactome pathways including ubiquitination and proteasome degradation in antigen processing (HSA-983168) and the immune system (HSA-168256), and tissue expression in embryos (BTO:0000379). Furthermore, KEGG pathway analysis revealed that the examined i-proteasomes and their differentially expressed genes were enriched in the following pathways: proteasome (hsa03050), bladder cancer (hsa05219), spinocerebellar ataxia (hsa05017), Huntington's disease (hsa05216), and prion disease (hsa05020).

**Table 4:** Functional enrichment in PSMB8/PSMB9 protein-coding gene loci network FDR, false discovery rate; GO, gene ontology; KEGG, Kyoto Encyclopedia of Genes and Genomes. a Number of proteins in the examined network/total number of proteins. b Log10 (observed/expected), describing the extent of the enrichment effect.

GO theme	Function	Count in network^a^		Strength^b^	FDR
Biological Process (GO):					
GO:0010499	Proteasome ubiquitin-independent protein catabolic process		45129	30713	3.30E-15
GO:0002479	Antigen processing and presentation of exogenous peptide via MHC class I, TAP-dependent		27607	2.37	4.26E-15
GO:0006521	Regulation of cellular amino acid metabolism process		23559	2.38	5.60E-13
GO:1902036	Regulation of hematopoietic stem cell differentiation		27211	2.31	1.05E-15
GO:0031145	Anaphase-promoting complex-dependent catabolic process		30498	2.26	1.21E-12
Cellular Component (GO):					
GO:0019773	Proteasome core complex, alpha-subunit		45024	3.04	3.46E-09
GO:0019774	Proteasome core complex, beta-subunit		44996	2.77	6.91E-06
GO:1990111	Spermatoproteasome complex		44962	2.94	0.00084
GO:0000932	P-body		31837	1.87	0.0011
KEGG Pathways (DAVID):					
hsa03050	Proteasome		15888	2.55	5.51E-15
Hsa05017	Spinocerebellar ataxia		5/135	1.91	3.60E-07
hsa05216	Huntington’s disease		5/298	1.65	6.92E-06
Hsa05020	Prion disease		5/265	1.61	4.87E-06
hsa05219	Bladder cancer		15008	1.83	0.0084
Reactome Pathways (GO):					
HSA-6803207	Regulation of activated PAK-2p34 by proteasome-mediated degradation		17715	2.5	2.41E-14
HSA-75815	Ubiquitin-dependent degradation of cyclin D		18445	2.48	1.67E-06
HSA-909733	interferon alpha/beta signaling		25235	1.8	0.0067
HSA-983168	Antigen processing: Ubiquitination & proteasome degradation		7/304	1.7	1.53E-10
HSA-168256	Immune system		20699	1	1.66E-08
Disease-gene associations (DISEASES):					
DOID:0050553	JMP syndrome		44959	3.34	0.0049
Tissue expression (TISSUES):					
BTO:0000379	Embryo		5/708	1.19	0.0134

## Discussion

This case-control study explored the common PSMB9 rs17587 G>A (p.R60H) and PSMB8 rs2071543 G>T (p.Q49K) SNPs as candidate biomarkers for UBC risk. Overall, our results showed that the rs17587 G>A SNP was strongly associated with a five-fold increased risk of UBC. Furthermore, the genotypic distributions significantly differed between cases and controls, except for the overdominant model, under all inheritance models. Results also suggest that the heterozygous PSMB9 rs17587 G/A genotype imparts little risk of UBC within the Saudi community. In contrast, no significant association was found between the PSMB8 rs2071543 G>T (p.Q49K) SNP and UBC susceptibility.

Studies have shown increased expression of proteasome subunit genes in several carcinomas. For example, genomic databases show upregulation of PSMB3, PSMA4, PSMD4, PSMA5, PSMD2, PSMA6, PSMA2, and PSMB7 in cancerous tissues when compared with normal tissues [22]. Although not adequate for sustaining proteostasis in neoplastic cells, these increases in expression are evident in serous ovarian carcinoma tissues compared with benign tumors or immortalized ovarian surface epithelia [23]. Analysis of genes expressed differentially in cancerous versus normal bladder tissues showed PSMB1 as a top-upregulated gene [24]. Several proteasome subunits' expression levels decrease after differentiation, with the most striking reduction in PSMB8 and PSMB9 [25].

Previous studies have indicated that the PSMB9 rs17587 G>A and PSMB8 rs2071543 G>T SNPs can cause functional alterations within cells. Also, these SNPs are associated with the development and occurrence of colorectal cancer, gastric carcinoma, and cervical cancer, as well as with the prognosis of patients with these cancers [12]. Another study reported that the PSMB9 rs17587 SNP was associated with the stage of non-small cell lung cancer, but no relationship was found between this SNP and lung cancer susceptibility [26]. Mishto et al. [27] reported that i-proteasome activity was significantly higher in individuals with the PSMB9 rs17587 G/G genotype than in those with the heterozygous G/A genotype, suggesting that heterozygosity is not associated with cancer risk. This report is consistent with the results of our study in Saudi individuals, as the heterozygous rs17587 G/A genotype was much less frequent in cases than in controls (15.4% versus 42.3%).

Wu et al. [17] conducted a meta-analysis of 19 published studies and reported that the PSMB9 rs17587 and PSMB8 rs2071543 SNPs were associated with increased cancer risk in the recessive model [17]. Furthermore, when these data were filtered by cancer type, individuals with the PSMB9 rs17587 SNP were more susceptible to gynecological cancers, while the PSMB8 rs2071543 SNP was associated with increased risk of gastrointestinal and gynecological cancers, especially in Asian populations [17,28]; the meta-analysis did not report results related to bladder cancer. Besides its immune-related role in UBC, PSMB9 serves a clear role in intracellular protein degradation in neurodegenerative diseases. A case-control study conducted to assess correlations between several genes and Parkinson's disease in a Chinese population showed that women carrying the PSMB9 rs17587 G/G genotype seemed to have an increased risk for Parkinson's disease, whereas men carrying the same genotype did not [29,30].

## Conclusions

Emerging data highlight an essential role for i-proteasome units in the risk of cancers, including bladder cancers, which might create novel opportunities for identifying diagnostic biomarkers and therapeutic interventions. The present study supports evidence that the PSMB9 rs17587G>A SNP, but not the PSMB8 rs2071543 G>T SNP, remarkably increases the risk of UBC in the Saudi population. However, these results should be interpreted with caution due to the complexity of UBC, as cancer heterogeneity and differences in ethnicity may influence the effects of these SNPs. The present study exploited a variety of bioinformatic analyses to identify the PSMB8/PSMB9 immunoproteasomes and their co-expressed genes, which may be supported for the early diagnosis and prevention of bladder carcinoma. Further studies using large datasets from UBC patient cohorts of different racial and ethnic backgrounds are necessary to decipher the impact of these findings on patient outcomes.
